# A machine learning approach applied to gynecological ultrasound to predict progression-free survival in ovarian cancer patients

**DOI:** 10.1007/s00404-022-06578-1

**Published:** 2022-05-09

**Authors:** Francesca Arezzo, Gennaro Cormio, Daniele La Forgia, Carla Mariaflavia Santarsiero, Michele Mongelli, Claudio Lombardi, Gerardo Cazzato, Ettore Cicinelli, Vera Loizzi

**Affiliations:** 1 Department of Biomedical Sciences and Human Oncology, Obstetrics and Gynecology Unit, University of Bari “Aldo Moro”, Piazza Giulio Cesare 11, 70124 Bari, Italy; 2 Interdisciplinar Department of Medicine, Obstetrics and Gynecology Unit, University of Bari “Aldo Moro”, Piazza Giulio Cesare 11, 70124 Bari, Italy; 3 Department of Breast Radiology, Giovanni Paolo II I.R.C.C.S. Cancer Institute, via Orazio Flacco 65, 70124 Bari, Italy; 4 Department of Emergency and Organ Transplantation, Pathology Section, University of Bari “Aldo Moro”, Piazza Giulio Cesare 11, 70124 Bari, Italy

**Keywords:** Machine learning, Ovarian cancer, Gynecological ultrasound, Progression-free survival

## Abstract

In a growing number of social and clinical scenarios, machine learning (ML) is emerging as a promising tool for implementing complex multi-parametric decision-making algorithms. Regarding ovarian cancer (OC), despite the standardization of features that can support the discrimination of ovarian masses into benign and malignant, there is a lack of accurate predictive modeling based on ultrasound (US) examination for progression-free survival (PFS). This retrospective observational study analyzed patients with epithelial ovarian cancer (EOC) who were followed in a tertiary center from 2018 to 2019. Demographic features, clinical characteristics, information about the surgery and post-surgery histopathology were collected. Additionally, we recorded data about US examinations according to the International Ovarian Tumor Analysis (IOTA) classification. Our study aimed to realize a tool to predict 12 month PFS in patients with OC based on a ML algorithm applied to gynecological ultrasound assessment. Proper feature selection was used to determine an attribute core set. Three different machine learning algorithms, namely Logistic Regression (LR), Random Forest (RFF), and K-nearest neighbors (KNN), were then trained and validated with five-fold cross-validation to predict 12 month PFS. Our analysis included n. 64 patients and 12 month PFS was achieved by 46/64 patients (71.9%). The attribute core set used to train machine learning algorithms included age, menopause, CA-125 value, histotype, FIGO stage and US characteristics, such as major lesion diameter, side, echogenicity, color score, major solid component diameter, presence of carcinosis. RFF showed the best performance (accuracy 93.7%, precision 90%, recall 90%, area under receiver operating characteristic curve (AUROC) 0.92). We developed an accurate ML model to predict 12 month PFS.

## Introduction

### Ovarian cancer

Ovarian cancer (OC) is the seventh-most-diagnosed cancer among women worldwide and the second-most-common gynecological malignancy. It represents appromixmately 14,000 deaths in 2020 in the US [1].

Up to 90% of ovarian cancers are epithelial ovarian cancer (EOC) types. OC has multiple cellular origins [2]. The term tubo-ovarian cancer is often used because OC can arise as an ovarian or fallopian-tube mass or primary peritoneal cancer [3].

Type I tumors (low-grade serous, mucinous, endometrioid, and clear cell) occurring in the ovary are less aggressive and are therefore more easly diagnosed at an early stage because they tend to grow slowly. Type II tumors (high-grade serous carcinomas (HGSC), undiffer-entiated carcinomas, and carcinosarcomas) may originate from the tubal and/or ovarian surface epithelium, and are more aggressive [4–6].

The absence of proper screening and diagnostic procedures to detect OC at an early stage as well as the rapid spread of disease through the peritoneal surface are leading factors in the OC lethality [7, 8]. Nowadays, there is a lack of an accurate protocol to identify high-risk patients.

Therefore, identifying tools for accurate screening and early diagnosis and prognosis of OC represents a currently unmet clinical need.

In addition, the role of ultrasound (US) in OC is evolving. US is a cheap, non-invasive and well-recognized image modality for diagnosis and evaluation of OC [9].

The International Ovarian Tumor Analysis (IOTA) group established a standardized lexicon that includes all appropriate descriptors and definitions of the sonographic appearance characteristic of normal ovaries and ovarian lesions. To simplify the sonographer’s assessment in differentiating benign from malignant adnexal masses, they also developed the Simple Rules classification system and the Assessment of Different Neoplasia in the Adnexa (ADNEX) model [10–16]. The Society of Radiologists in Ultrasound consensus statement [17, 18] and the Gynecologic Imaging Reporting and Data System, also known as GI-RADS [19], are other proposed systems for the characterization and management of ovarian masses (OM) [20].

In 2018, the Ovarian-Adnexal Reporting and Data System (O-RADS) created a risk stratification classification for consistent follow-up and management in clinical practice [21].

But quickly, a simple description of the tumor and of its extension may not be sufficient. The application of precision medicine could help answering a question about early response to treatment, best timing for surgery, prognosis or molecularly targeted drug.

### Machine Learning

In a growing number of social and clinical scenarios, machine learning (ML) is emerging as a promising tool for the implementation of complex multi-parametric decision-making algorithms [22, 23]. In that sense, a ML approach is a potential gamechanger [24]. In fact, in addition to detecting linear patterns in analyzed data, it can unravel complex non-linear relationships between patient attributes that cannot be solved by traditional statistical methods, merging them to produce a prediction or a probability for a given outcome [22, 25, 26].

ML is a step toward precision medicine, leading to improved patient profiling and personalized treatment. Supervised ML algorithms have been shown to be effective in predicting treatment responses and disease progression in patients affected with heterogeneous diseases [27, 28].

Regarding OC, despite the standardization of features that can support the discrimination of ovarian masses into benign and malignant, there is the lack of accurate predictive modeling based on US examination for PFS.

## Materials and methods

In this retrospective observational study, we analyzed consecutive patients with EOC who were followed in a tertiary center from 2018 to 2019.

Demographic features (age), clinical characteristics (parity, menopause, CA-125 value, genetic mutation state, treatment) were collected as well as information about surgery (surgical procedures, residual tumor) and post-surgery histopathology (histotypes, grading, FIGO stage). Additionally, we recorded data about transvaginal and/or transabdominal US examinations according to IOTA classification (unilateral lesion, side, largest diameter of lesion, type of tumor, echogenicity of cyst fluid in tumors, color score, diameter of largest solid component, shadows, ascites, carcinosis, subjective assessment).

Our study aimed to realized a tool to predict 12 month PFS in patients with OC based on a ML algorithm applied to gynecological ultrasound assessment.

In total, the original database included n. 64 patients and n. 22 variables.

Appropriate feature selection was used to determine an attribute core set (see Supplementary Materials for further details).

This study followed STARD guidelines [29] and the TRIPOD statement [30].

The ML algorithms were aimed at forecasting PFS at 12 month follow-up.

Student’s *t* test for paired samples or Wilcoxon matched-pair signed-rank test were used as appropriate to identify difference between continuous variables between different observation periods. McNemar’s test was used to identify the difference among dummy variables between.

The attribute core set used to train the algorithms was determined using a recursive feature elimination (RFE) wrapper based on a decision tree algorithm with extreme gradient boosting (XGBoost) [31]; in brief, this algorithm automatically selects from all the recorded attributes (n. 23) the best number of features on their importance for the given outcome predictions (PFS at 12 months). Feature selection can counteract overfitting problems and improve classification performance. RFE method is one of the commonly used feature selection methods for small samples problems [32–34] (For further details about RFE see Supplementary Materials).

The entire analysis was implemented in a Python 3.6 environment using scikit-learn (ver.0.22.1) and XGBoost (ver. 1.1.0) libraries [31, 35]. After z-score normalization, we performed a Bayesian ridge conditional ridge imputation [36] for missing data. The latter method proved to be the most accurate method of imputation for obstetrics and gynecology datasets [37] (see Supplementary Materials for further details).

Three different classifiers, both linear and non-linear, were trained and cross-validated with five-fold cross-validation using the core set of attributes recovered from the RFE to predict 12 month PFS.

While logistic regression (LR) was almost always the algorithm of choice to find independent predictors in multivariate models, it must be noticed that the study hypotheses were usually based on the unrealistic assumption that the association between the prognostic factors and clinical outcomes is direct and isolated. In contrast, LR is not suitable for the modeling of non-independent variables. For this reason, along with usual LR, for linear modeling, we used the non-parametric K-nearest neighbors (KNN) and random forest (RFF) [36] algorithms. The latter models have recently been shown to accurately predict important outcomes for woman’s health, even in the presence of non-linear patterns in data [38–40]. Furthermore, we choose RFF because there is evidence of accurate performance in case of unbalanced data, which is often the case of clinical datasets [41]. We also ran RFF using cost-sensitive training (using the argument class weight = “balanced” in scikit-learn) to try to overcome unbalanced class issue.

A repeated grid-search with cross-validation was used for optimal hyperparameter tuning to maximize the classifiers’ performance [42] (See Supplementary Material for hyperparameter fine-tuning).

For each classifier, we plotted ROC curves, and then area under receiver operating characteristic curve (AUROC) was determined.

Then, based on the optimal probability cut-off (Youden’s Index) [43] classifiers’ performance was compared with the following metrics:

- $${\text{Accuracy}}\,{ = }\,\frac{{{\text{true}}\,{\text{positivies}}\,{ + }\,{\text{true}}\,{\text{negatives}}}}{{{\text{true}}\,{\text{positivies}}\,{ + }\,{\text{true}}\,{\text{negatives}}\,{ + }\,{\text{false}}\,{\text{positives}}\,{ + }\,{\text{false}}\,{\text{negatives}}}}{,}$$
- $${\text{Recall}}\,\left( {{\text{True}}\,{\text{Positive}}\,{\text{Rate}}\,\left( {{\text{TPER}}} \right)\,} \right) { = }\, \frac{{{\text{true}}\,{\text{positives}}}}{{{\text{true}}\,{\text{positivies}}\,{ + }\,{\text{false}}\,{\text{negatives}}}}{,}$$
- $${\text{Precision}}\,{ = }\,\frac{{{\text{true}}\,{\text{positives}}}}{{{\text{true}}\,{\text{positives}}\,{ + }\,{\text{false}}\,{\text{positives}}}}$$

In general, a classification model forecasts a binary outcome for a given observation and class. In the process of predicting, a model may output the probability of an observation belonging to each possible class. This case allows some flexibility in the way predictions are interpreted and presented, allowing the choice of a threshold, such as the afore-mentioned Youden’s index [44].

For a model to be reliable, the estimated class probabilities should reflect the true underlying probability of the sample. To check these assumptions, a diagnostic calibration curve for the candidate best classifier was also plotted [44].

The study was conducted in accordance with the Declaration of Helsinki, and the protocol was approved by the Scientific Board University of Bari, Bari, Italy. All patients had signed a consent to use the data in scientific purposes.

## Results

Our analysis included n. 64 patients with diagnosis of EOC. Demographic and clinical characteristics, information about surgery procedures, post-surgery histopathology and US features are outlined in (Table 1).

**Table 1:** Cohort characteristics. Variables of the original dataset (*n*.22) are listed in bold

Age at diagnosis (years), mean ± SD	54.1 ± 14.9 years
Parity, median (IQR)	1 (0–2)
Menopause,* n*. (%)	28/64 (43.7%)
CA-125 (U/mL), mean ± SD	828.25 (± 2018.82)
Genetic mutation state	4/64 (6.25%)
BRCA1m,* n*. (%)	
BRCA2m,* n*. (%)	4/64 (6.25%)
BRIP1m,* n*. (%)	2/64 (3.12%)
Unilateral tumor,* n*. (%)	34/64 (53.1%)
Side,* n*. (%)	
Right	16/64 (25%)
Left	18/64 (28.1%)
Middle	30/64 (46.9%)
Largest diameter of lesion (mm), mean ± SD	113.6 ± 57.6
Type of tumor,* n*. (%)	
Unilocular	0
Unilocular-solid	8/64 (12.5%)
Multilocular	2/64 (3.1%)
Multilocular-solid	28/64 (43.7%)
Solid	26/64 (40.7%)
Echogenicity of cyst fluid in tumors not classified as solid,* n*. (%)	
Anechoic	22/64 (34.4%)
Ground glass	6/64 (9.4%)
Low level	10/64 (15.6%)
Color Score,* n*. (%)	
1	8/64 (12.5%)
2	6/64 (9.4%)
3	18/64 (28.1%)
4	32/64 (50%)
Diameter of largest solid component (mm), mean ± SD	71.1 ± 45.1
Shadows,* n*. (%)	8/64 (12.5%)
Ascites,* n*. (%)	18/64 (28.1%)
Carcinosis,* n*. (%)	20/64 (31.2%)
Diagnosis on basis of subjective assessment, n. (%)	
Benign	8/64 (12.5%)
Malignant	56/64 (87.5%)
Surgery,* n*. (%)	
Open surgery	49/64 (76.5%)
Laparoscopy	15/64 (23.5%)
Residual Tumor,* n*. (%)	
R0	48/64 (75%)
R1 or R2	16/64 (25%)
Histotypes,* n*. (%)	
High-grade serous	42/64 (65.6%)
Endometrioid	10/64 (15.6%)
Clear cell	8/64 (12.5%)
Mucinous	4/64 (6.3%)
Grading,* n*. (%)	
G1	12/64 (18.7%)
G2	2/64 (6.3%)
G3	48/64 (75%)
FIGO Stage,* n*. (%)	
I	22/64 (34.4%)
II	2/64 (3.1%)
III	26/64 (40.6%)
IV	14/64 (21.9%)
Treatment,* n*. (%)	
No treatment	6 /64 9.4%)
Neoadjuvant therapy	24/64 (37.5%)
Adjuvant chemotherapy	
Paclitaxel–Carboplatin	24/64 (37.5%)
Paclitaxel–Carboplatin–Bevacizumab	2/64 (3.1%)
Paclitaxel–Carboplatin–Parp inhibitor	8/64 (12.5%)

Patients had a mean age (± SD) of 54.1 ± 14.9 years at diagnosis and n. 28/64 (43.7%) were menopausal patients. CA-125 median value was 828.25 (± 2018.82) U/mL. Four out of 64 (6.25%) women had BRCA1 mutation, n. 4/64 (6.25%) women had BRCA2 mutation and n. 2/64 (3.12%) women had BRIP1 mutation.

Concerning US characteristics, n. 34/64 (53.1%) patients had a unilateral mass and the median greatest diameter was 113.6 ± 57.6 mm. The most common tumor type was multilocular-solid (28/64 (43.7%)), followed by solid (26/64 (40.7%)), unilocular-solid (8/64 (12.5%)), and multilocular (2/64 (3.1%)) masses. The median diameter of the largest solid component was 71.1 ± 45.1 mm. The most common echogenicity of cyst fluid was anechoic (22/64 (34.4%)), followed by low level echogenicity in n.10/64 (15.6%) and ground glass echogenicity in n. 6/64 (9.4%). Most of these tumors showed intense vascularity on color Doppler examination (32/64 (50%)) and n. 18/64 (28.1%) moderate vascularity. Based on the subjective assessment by the original US examiner, n. 56/64 (87.5%) masses were classified as malignant and n. 8/64 (12.5%) as benign tumors. Ultrasonographic evaluation revealed ascites in n. 18/64 (28.1%) and carcinosis in n. 20/64 (31.2%). Only n. 8/64 (12.5%) revealed shadows.

Concerning the surgical procedure, n. 49/64 (76.5%) underwent open surgery and n. 15/64 (23.5%) underwent laparoscopy. Forty eight out of 64 (75%) presented no residual tumor; n. 16/64 (25%) presented microscopic (R1) or macroscopic (R2) residual tumor.

On histopathological analysis, histotypes were n. 42/64 (65.6%) high-grade serous carcinoma, n. 10/64 (15.6%) endometrioid, n. 8/64 (12.5%) clear cell and 4/64 (6.3%) mucinous. Grade was G1 in n. 12/64 (18.7%), G2 in n. 2/64 (6.3%), G3 in n. 48/64 (75%). Most tumors were FIGO Stage III (26/64 (40.6%)), followed by FIGO stage I (22/64 (34.4%)) and FIGO stage IV (14/64 (21.9%)). Only n. 2/64 (3.1%) had a FIGO stage II.

Twenty four out of 64 (37.5%) were treated with neoadjuvant chemotherapy with paclitaxel–carboplatin, n. 24/64 (37.5%) with adjuvant chemotherapy with paclitaxel–carboplatin, n. 8/64 (12.5%) adjuvant chemotherapy with paclitaxel–carboplatin and parp inhibitor and n. 2/64 (3.1%) paclitaxel–carboplatin and bevacizumab. Six out of 64 (9.4%) required no treatment. 12-month PFS was achieved by 46/64 patients (71.9%, unbalanced classes).

**Fig. 1:**
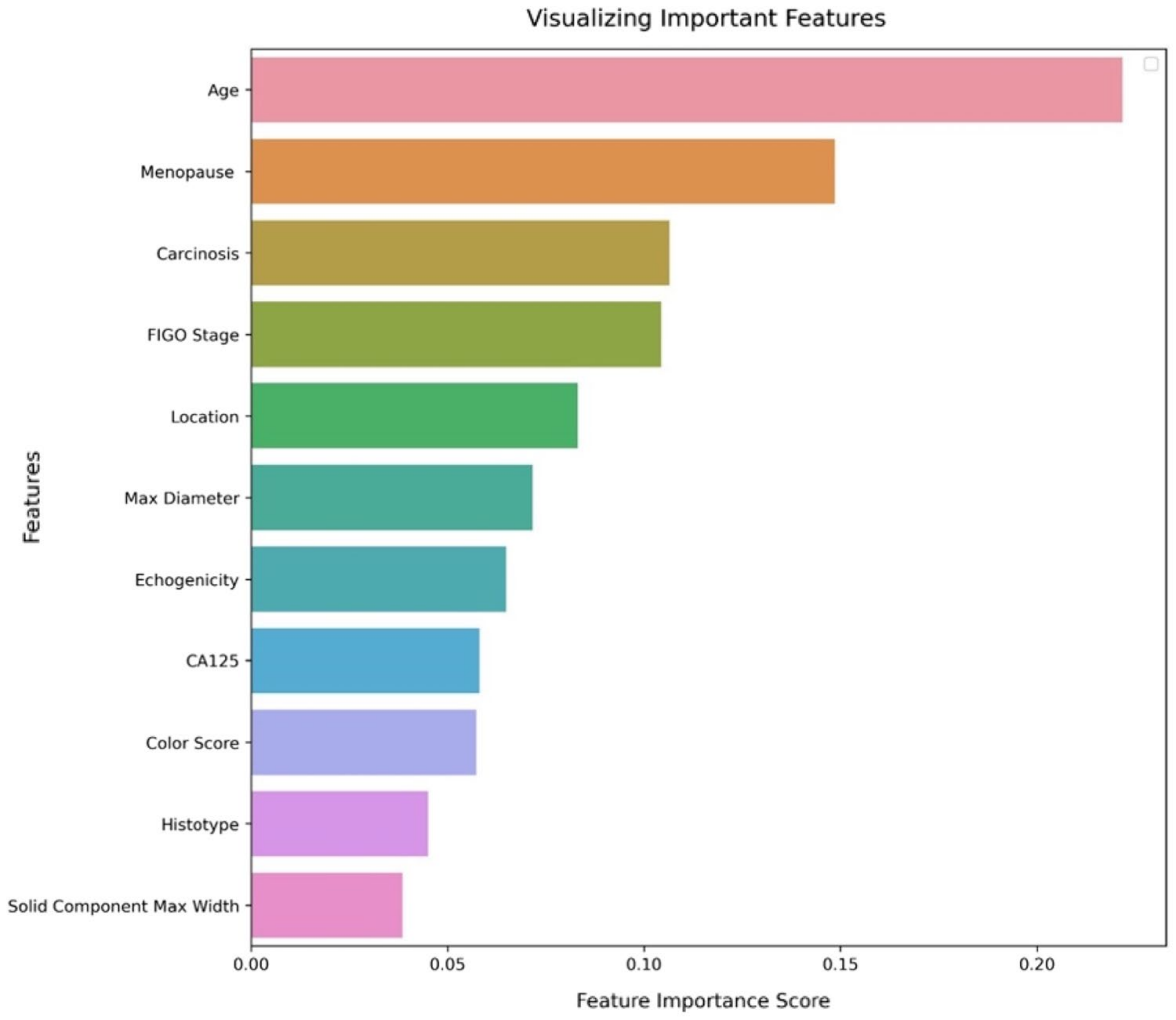
Feature importance of the attribute coreset

**Fig. 2:**
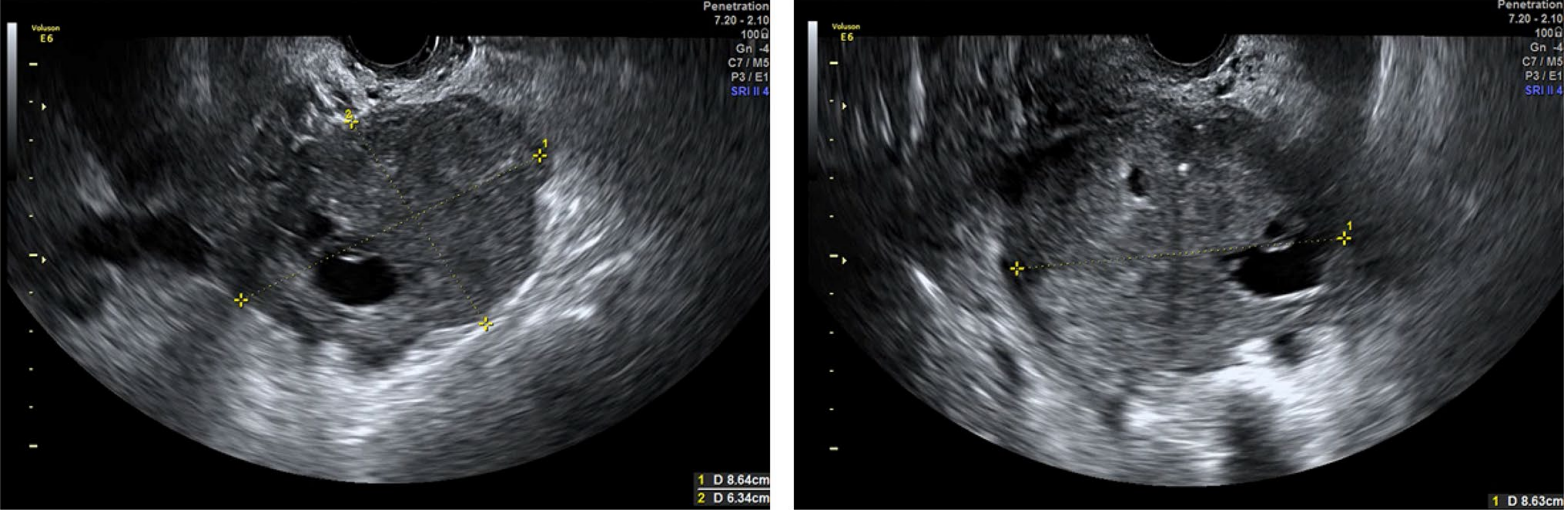
Size lesions measurement. The sizes of the lesion are measured as the largest three diameters (in mm) in two perpendicular planes. The largest diameter was found to be one of the most important features to predict PFS

**Fig. 3:**
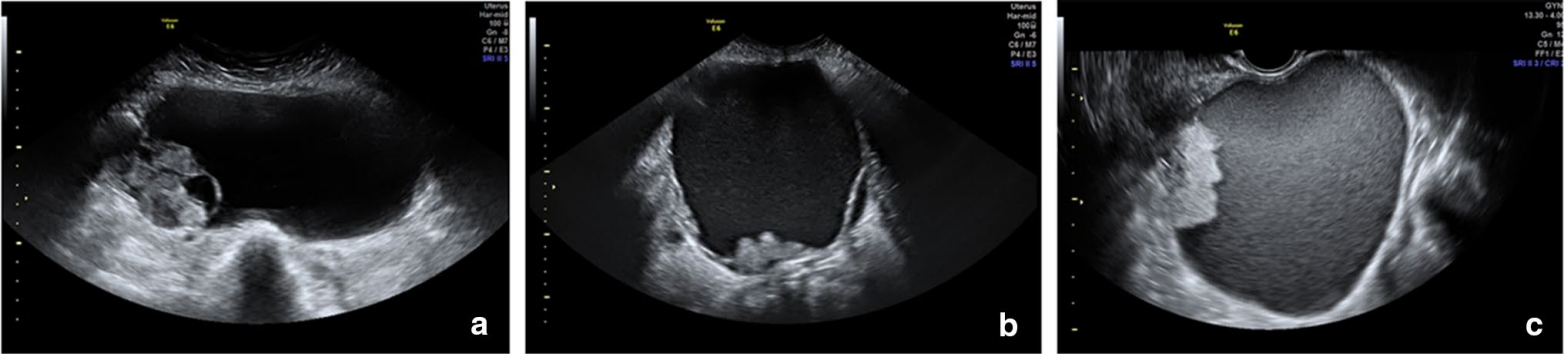
Echogenicity of cyst fluid. The echogenicity of cyst fluid in tumors not classified as solid is described as anechoic (Panel **a**, low level (homogeneous low level echogenic) (Panel **b**) or ground glass (homogeneously dispersed echogenic cystic contents) (Panel **c**)

**Fig. 4:**
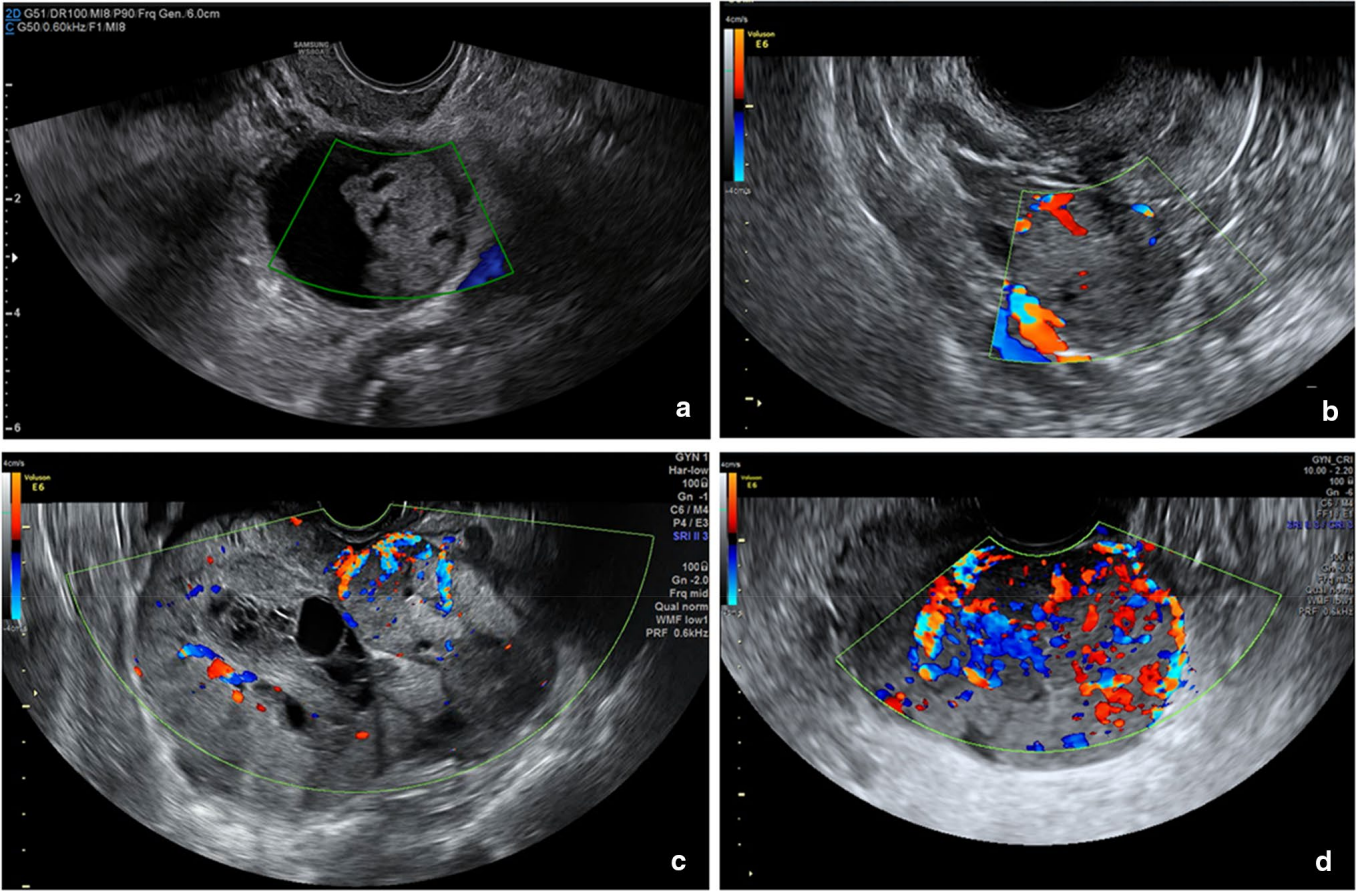
Assessment of blood flow. The assessment of blood flow is a subjective assessment evaluated with a color scale. Panel **a** Color score 1: no flow, Panel **b** Color score 2: minimal flow, Panel **c** Color score 3: moderate flow, Panel **d** Color score 4: intense flow. The color score evaluation was found to be one of the most important features to predict PFS

**Fig. 5:**
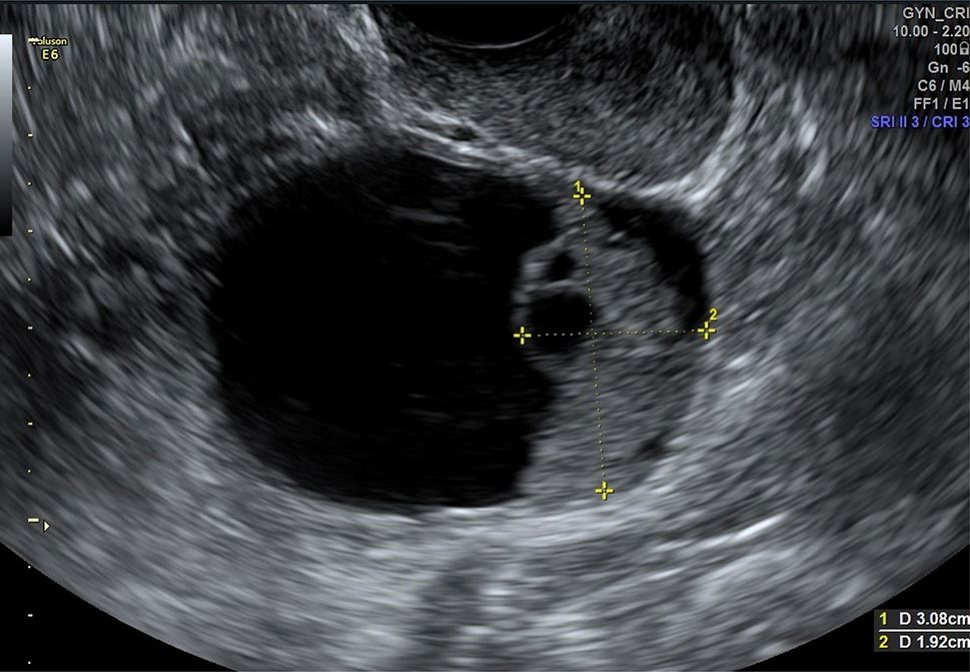
Major solid component diameter measurement. The largest solid component in a cystic solid tumors is measures separately with the assessment of two or three diameters in two perpendicular planes. The largest solid component was found to be one of the most important features to predict PFS

**Fig. 6:**
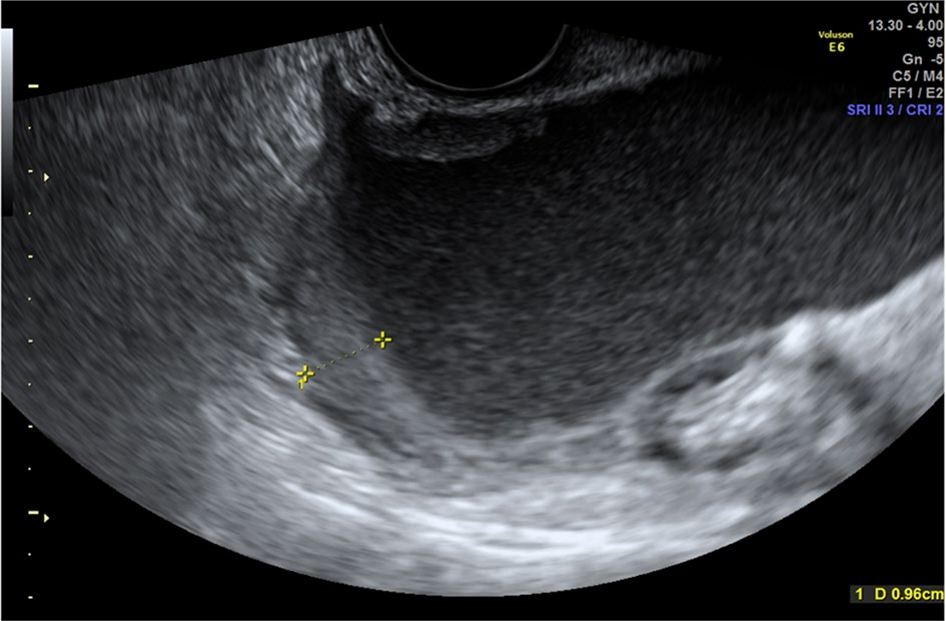
Ultrasound finding of carcinosis. Ultrasound assessment of carcinosis was found to be one of the most important features to predict PFS

As detailed in (Fig. 1), RFE retrieved an attribute core set used to train machine learning algorithms including age, menopause, CA-125 value, histotype, FIGO stage and US characteristics, such as major lesion diameter (Fig. 2), side, echogenicity (Fig. 3), color score (Fig. 4), major solid component diameter (Fig. 5), presence of carcinosis (Fig. 6).

The attribute core set used to train machine learning algorithms is reported in (Fig. 1). RFF showed an accuracy of 0.93, AUROC 0.92.

The final dataset had a dimensionality of 64 columns × 12 rows (n.11 selected attributes plus n. 1 target class (PFS at 12 months, as above mentioned).

As reported in (Table 2), at optimal cut-off (Youden’s index), RFF (n. estimators = 500, depth = 5) showed the best performance (accuracy 93.7%, precision 90%, TPR 90%, AUROC 0.92), outperforming LR (accuracy 82%, precision 80.1%, TPR 84.1%, AUROC 0.81), and KNN (n. of neighbors = 5) (accuracy 73.6%, precision 76.5%, TPR 83.3%, AUROC 0.69).

**Table 2:** Algorithms Performance

	Youden’s index cut-off	Accuracy (%)	TPR (%)	Precision (%)	AUROC
LR	0.64	82	84.1	80.1	0.81
RFF	0.77	**93.7**	**90**	**90**	**0.92**
KNN	0.68	73.6	83.3	76.5	0.69

Algorithm with the best performance on five-fold cross-validation is indicated in bold. Accuracy, Recall, Precision and AUROC for RFF, were significantly better than other algorithms’ ones

*AUROC* area under receiver operating characteristics curve, *LR* logistic regression, *KN*N K-nearest neighbors, *RFF* random forest *TPR* true positive rate

**Fig. 7:**
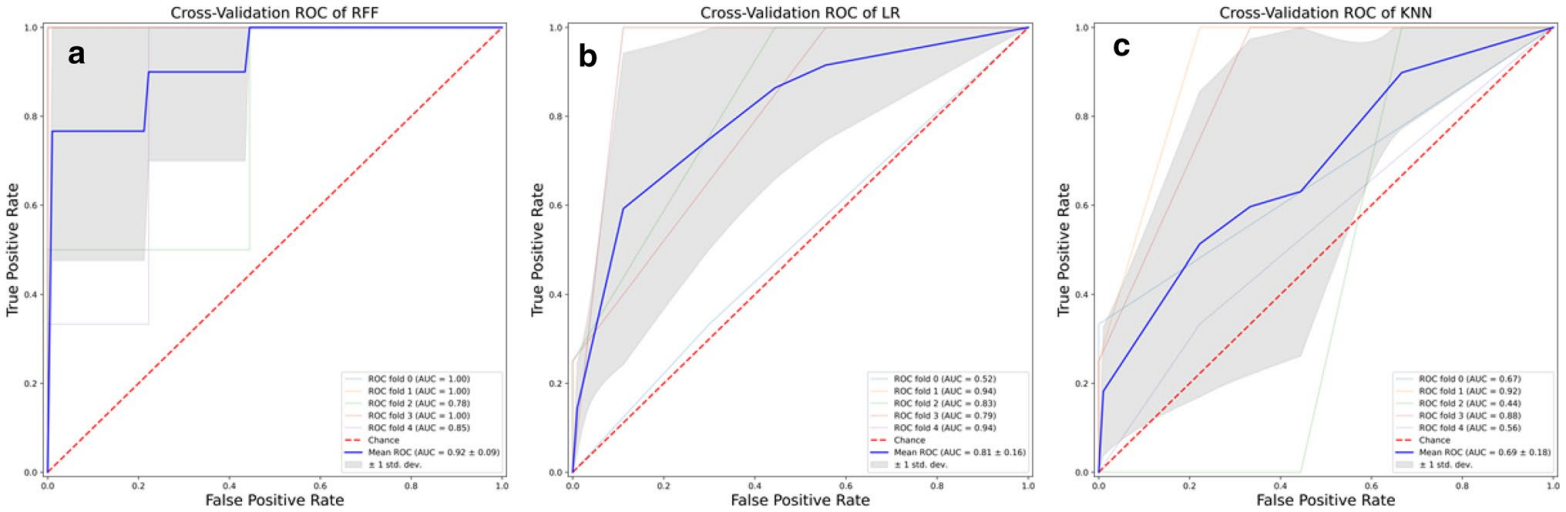
Receiver operating characteristics curve for Random Forest (box (**A**)), Logistic Regression (box (**B**)) and K-nearest neighbors (box (**C**)) models

In (Fig. 7), ROC curve for RFF (box A), LR (box B) and KNN (box C) models was reported.

**Fig. 8:**
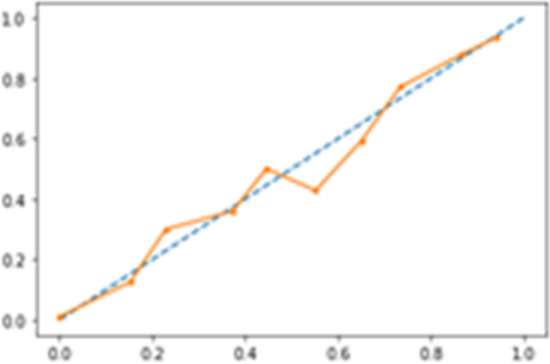
Calibration diagnostics for RFF model. 12 month PFS roughly happened with an observed relative frequency consistent with the forecast value, showing good calibration

In (Fig. 8) calibration diagnostic has been plotted for RFF; PFS roughly happened with an observed relative frequency consistent with the forecast value, showing an acceptable calibration curve. We would expect the match between predicted frequencies and observed frequencies to increase with a larger dataset.

We also reported the Odds ratios for the LR model for the interpretation of core set covariate associations in (Table 3).

**Table 3:** Odds ratios for the logistic regression model (outcome event: relapse within 12 months)

	OR	95% CI
Age at diagnosis	1.11	1.05–1.18
Menopause	22.67	4.52–113.46
Ca125	1.001	1.000069–1.001946
Side	0.40	0.25–1.25
Histotype	1.47	1.17–1.83
Echogenicity of cyst fluid	0.20	1.10–1.90
Color score	1.53	0.82–2.84
Largest diameter of lesion	0.99	0.98–1.00
Carcinosis	9.5	2.74–32.88
Figo stage	7.39	2.27–24.07
Solid tumor	1.01	0.99–1.01

## Discussion

The keystone of survival analyses in cancer research has historically been Cox proportional hazard regression model, being a surrogate for estimating treatment efficacy and safety. This model is based on the assumption of linear association. However, many clinicopathologic features show a non-linear association in medicine [45].

The ML approach has recently brought an unprecedented growth of applications to medical imaging.

In the study of OC, since 1999, artificial neural networks [46, 47] have been applied to classify US image into benign and malignant, but image features were manually measured and provided by the investigators.

In 2015, Kazendar et al. [48] developed a fully automatic ML classifier stratifying US images as benign or malignant masses with an accuracy of 77% when images were enhanced with a Local Binary Pattern operator.

Recently, due to the wide availability of digital medical images and the technical advances in hardware and software, ML has also been applied in conjunction with radiomic analysis.

In a study by Chiappa et al. [49], ML and radiomics were applied to transvaginal ultrasonography (TUS) to implement a decision support system (DSS) for predicting the risk level of malignancy of OM.

The DSS was based on a set of three radiomic ML models, named as solid masses, cystic masses and mixed masses. These radiomic models were integrated with information about presence/absence of acoustic shadows and serum CA-125 level, considering two different thresholds according to menopausal status.

This addition integrates the malignancy risk predicted by each of the three TUS radiomic models.

The DSS was based on TUS imaging and serum CA-125 level and showed 91% accuracy, 100% sensitivity, and 80% specificity in independent tests.

Martinez-Mas et al. [50] realized a ML algorithms aimed to perform the automatic categorization of OC from US images. They analyzed 348 images. For each patient case and US image, its input features were previously extracted using Fourier descriptors calculated over the Region Of Interest (ROI). Then, four ML algorithms were considered to perform the classification stage: KNN, Linear Discriminant (LD), Support Vector Machine (SVM) and Extreme Learning Machine (ELM). LD, SVM and ELM reported more than 85% accuracy.

Regarding ML applications in the clinical management of OC patients, Hwangbo et al. [51] aimed to develop ML models predicting platinum sensitivity in patients with HGSC. Using the stepwise selection method, based on the AUC values, six variables associated with platinum sensitivity were selected: age, initial serum CA-125 levels, neoadjuvant chemotherapy, pelvic lymph node status, pelvic tissue involvement other than uterus and tubes, and small bowel and mesentery involvement. Based on these variables, predictive models were constructed using four ML algorithms, LR, RFF, SVM and deep neural network. Evaluation of model performance using the five-fold cross-validation method identified the LR-based model as the best for identification platinum-resistant cases. Therefore, they developed a web-based nomogram adapting the LR model results for clinical utility.

Also attempting to improve treatment choices of OC patients, Shannon et al. [52] developed a ML tool to identify predictive molecular markers for cisplatin chemosensitivity.

CYTH3, GALNT3, S100A14, and ERI1 were the four potential biomarkers identified. Validation was performed on a cohort of n. 50 patients who underwent surgery followed by adjuvant carboplatin. Predictive models were established to predict chemosensitivity. The four biomarkers were also evaluated for their ability to prognosticate overall survival (OS) in three OC microarray expression datasets from The Gene Expression Omnibus. The extreme gradient boosting (XGBoost) algorithm was selected for the final model to validate the accuracy in an independent validation dataset (*n* = 10). CYTH3 and S100A14, followed by nodal stage, were the most important features. The signature of the four genes had a comparable prognosis to clinical information for two-year survival.

To date, only few studies attempted to apply ML to ultrasound evaluation of adnexal masses to predict benign or malignant histology.

On the other hand, some authors applied ML using only clinical and laboratory data to predict treatment response. To our best knowledge, this is the first ML algorithm basing on clinical, surgical, histophalogical and US features to predict PFS in patients diagnosed with OC.

The variables identified by the RFE as the attribute core set to predict the PFS had been already studied in literature.

In our cohort, age and menopausal status were negatively associated with PFS (Table 3). Consistently, Okunade et al. reported that age ≤ 55 years was an independent predictor of improved PFS [53]. In the study of Trifanescu et al., in premenopausal women, PFS was significantly higher than in post-menopausal ones [54].

In clinical practice, residual tumor is regarded as the most important factor for PFS [53]. Patients with absence of residual tumor after primary debulking surgery or interval debulking surgery have an increased PFS and OS rates compared to patients with residual tumor [55]. However, in our study, this was not identified by ML as a predictor of prognosis*.* Of note running a LR for inferential purpose, residual tumor was found associated with PFS (OR 3.04, 95% CI 1.62–4.46, data not shown). Additionally, residual tumor was strongly correlated with high FIGO Stage in our cohort (Cramer’s *V* = 0.91, data not shown). In this regard, on building a XGBoost-based RFE wrapper, it must be noticed that such multicollinearity is auto-handled and algorithm only keeps one of autocorrelated attributes for splitting trees [56]. This might explain why residual tumor was not included in the attribute core set.

The main limitation of our study is the low sample size, which in fundamental in ML research. Neverdless RFF as proven robust in previous studies with low or similar sample size [23]. To be adopted in clinical practice, the algorithm will need extensive external validation on larger prospective cohorts.

In gynecologic oncology, ML is a step toward precision medicine, leading to an improved patient profile and personalized treatment.

This model could be applied at the time of diagnosis to predict 12 month PFS in patients with OC. Ultrasound is a simple, non-invasive and inexpensive examination. The creation of a ML approach applied to gynecological ultrasound could allow to personalize the follow-up, stratifying patients according to the predicted PFS, intensifying the prescription of instrumental examinations in high-risk patients and reducing the request in low-risk patients.

This algorithm requires few easy-to-collect attributes. Further studies are needed to assess the potential of ML algorithms in routine gynecologic care.

## Data Availability

Data are not freely available due to local Ethics Committee privacy issues. Authors will consider data sharing upon specific request to local Ethics Committee.
